# Quality standards for child and adolescent mental health in primary care

**DOI:** 10.1186/1471-2296-13-51

**Published:** 2012-06-06

**Authors:** Kapil Sayal, Myanthi Amarasinghe, Sarah Robotham, Caroline Coope, Mark Ashworth, Crispin Day, Andre Tylee, Emily Simonoff

**Affiliations:** 1 Section of Developmental Psychiatry, University of Nottingham, Nottingham, NG7 2UH, UK; 2 King’s College London (Institute of Psychiatry), London, UK; 3 Department of Primary Care and Public Health Sciences, King’s College London School of Medicine, London, UK

## Abstract

**Background:**

Child and adolescent mental health problems are common in primary healthcare settings. However, few parents of children with mental health problems express concerns about these problems during consultations. Based on parental views, we aimed to create quality of care measures for child and adolescent mental health in primary care and develop consensus about the importance of these quality standards within primary care.

**Methods:**

Quality Standards were developed using an iterative approach involving four phases: 1) 34 parents with concerns about their child’s emotional health or behaviour were recruited from a range of community settings including primary care practices to participate in focus group discussions, followed by validation groups or interviews. 2) Preliminary Quality Standards were generated that fully represented the parents’ experiences and were refined following feedback from an expert parent nominal group. 3) 55 experts, including parents and representatives from voluntary organisations, across five panels participated in a modified two-stage Delphi study to develop consensus on the importance of the Quality Standards. The panels comprised general practitioners, other community-based professionals, child and adolescent psychiatrists, other child and adolescent mental health professionals and public health and policy specialists. 4) The final set of Quality Standards was piloted with 52 parents in primary care.

**Results:**

In the Delphi process, all five panels agreed that 10 of 31 Quality Standards were important. Although four panels rated 25–27 statements as important, the general practitioner panel rated 12 as important. The final 10 Quality Standards reflected healthcare domains involving access, confidentiality for young people, practitioner knowledge, communication, continuity of care, and referral to other services. Parents in primary care agreed that all 10 statements were important.

**Conclusions:**

It is feasible to develop a set of Quality Standards to assess mental healthcare provision for children and adolescents seen within primary healthcare services. Primary care practitioners should be aware of parental perspectives about quality of care as these may influence help-seeking behaviours.

## Background

Between 10-20% of children and adolescents have significant difficulties with their emotional health or behaviour resulting in functional impairment [1]. These can affect their future development and outcomes in terms of their mental health, education, employment and relationships. Although children with mental health problems are more likely to be seen in primary care than by specialist mental health services and are regular attenders in this setting, less than one-quarter of children meeting criteria for caseness are presented to primary care with mental health problems [2,3]. Over the past decade, there has been a strong international emphasis on improving the quality of healthcare. The Institute of Medicine in the United States has specified patient-centred and equitable care as key components of quality health care [4]. UK policy initiatives over this time have also aimed to improve the quality of care and reduce inequities in access to care. These have stressed the need to enhance the quality of healthcare services and develop consumer-derived measures of service quality [5]. Although the UK National Service Framework for Children [6] stated that primary care professionals should be competent to recognise, support and refer children with mental health problems, it focused on recommending standards of care rather than specifying quality measures. Specific quality initiatives within primary care such as the Quality and Outcomes Framework have also neglected child and adolescent mental health. More recently, the National Institute of Health and Clinical Excellence (NICE) has been tasked with developing quality standards for the National Health Service. In addition, the Commissioning for Quality and Innovation (CQUIN) payment framework now provides a vehicle for local service commissioners to make funding allocations to services contingent upon achieving agreed quality improvement targets. Although there has been some research assessing the patient experience and developing quality standards in primary care settings for adults with mental health problems [7,8], there has been a dearth of similar work for children.

There is little available evidence for the optimal organisation of primary healthcare services for children and adolescents with mental health problems. There is also a lack of practice-level measures to assess the quality of care provided for these children within primary care. The development of suitable quality of care measures has implications for service development and organisation, clinician training and practice-level interventions that aim to improve quality of care and outcomes for children. This study uses a parent/caregiver led approach to develop a measure for assessing and improving the quality of care for children with mental health problems. Based on parental views, we aimed to develop parent/caregiver derived quality standards for primary care child and adolescent mental health and to develop consensus about the importance of these quality standards within primary care.

## Methods

**Figure 1:**
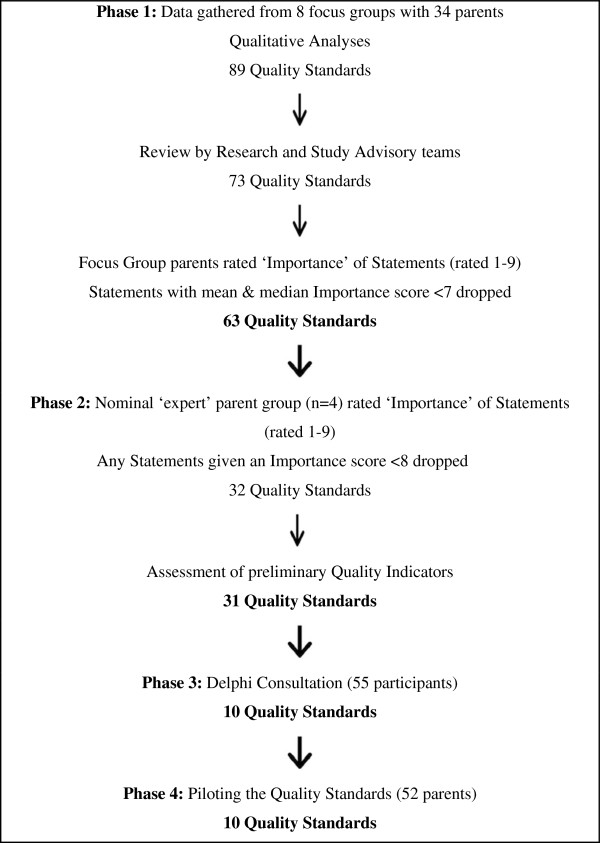
Flow Chart of the 4 phases in the development of the Quality Standards.

The study took place in South London and involved four phases (see Figure 1). Written informed consent for participation in the study was obtained from participants. Ethical approval for the study was received from the Joint South London and Maudsley and the Institute of Psychiatry NHS Research Ethics Committee.

### Phase 1: Focus groups and development of quality standards

From a wide range of community settings, we recruited parents of children aged 2–15 years who had concerns about their child’s emotional health or behaviour but who were not currently under the care of specialist Child and Adolescent Mental Health Services (CAMHS) [9]. This was done in order to focus on their use of primary care services and barriers to receiving specialist services [10,11], perceptions of previous help-seeking experiences, access and communication issues when using services, and suggestions for improving primary care services. The selection of parents (n = 34) from different settings aimed to reflect a wide range of child age and possible routes of initial presentation in order to assist with the development of generalisable Quality Standards for primary care child and adolescent mental health. Most children had clinically significant symptoms and parental concerns reflected behavioural, emotional, learning and developmental difficulties. In summary, 8 initial focus group discussions were followed by validation groups or semi-structured interviews held in order to clarify the information gathered and to obtain further ideas on how to improve access to and standards of care for child and adolescent mental health in primary care. Full details about the focus groups are presented elsewhere [9].

A coding framework was developed to generate preliminary statements that reflected the parents’ verbatim. These statements aimed to highlight best practice in terms of access to services and service delivery within primary care (i.e. reflecting Quality Standards). The initial statements were reviewed and refined over three iterations by two researchers and checked for overlap with other statements and against the verbatim data. A total of 89 Quality Standards that fully represented the parents’ experiences were then reviewed by the wider research and Study Advisory teams to assess face validity, improve clarity and minimise duplication. The research team reflected backgrounds in primary care and child and adolescent psychology and psychiatry. The Study Advisory team consisted of representatives from the voluntary sector (Young Minds and the National Attention Deficit Disorder Information and Support Service), children and young people's services (Connexions, a service providing information and advice for young people) and CAMHS (Patient and Public Involvement Co-ordinator). Based on this feedback, 16 standards were removed. Parents from the focus groups were then asked, by post, for their feedback on the remaining 73 Quality Standards and to rate them for ‘clarity’ and ‘importance’. Based on their responses, 10 standards with mean and median ‘importance’ scores of <7 (on a 1–9 scale with 1 being ‘not important’ and 9 being ‘important’) were removed and six were re-written based on equivalent ‘clarity’ scores.

### Phase 2: Nominal group of “expert parents” and feedback on quality standards and indicators

Four different parents with experience of using both primary and secondary care services for their child’s mental health were recruited and asked to rate the 63 Quality Standards on ‘clarity’ and ‘importance’. Following this, they participated in a focus group to discuss their ratings and to re-rate the Quality Standards on ‘importance’. Based on this, any Quality Standard that was scored <8 (on a scale of 1–9) by any participant was removed. This led to 32 Quality Standards being retained. For each Quality Standard, the parents also suggested several practical ways in which this could be measured in practice i.e. Quality Indicators [12].

These parent-derived Quality Standards were grouped together to reflect four conceptual domains: a) practice level factors (including advertising, information and confidentiality); b) consultation factors (skills, knowledge, awareness and communication); c) health visitors (primary healthcare and community specialist nurses primarily focussing on children aged 5 years or less and their families); and d) further services (access, referrals and ideal services).

The Quality Standards and the preliminary Quality Indicators were introduced to user group representatives, clinicians (from CAMHS and primary care), and service managers for preliminary feedback about their acceptability in practice and face validity. This was done in order to further assess and refine the importance of the Quality Standards and also to select the most feasible Quality Indicator for each standard. Based on their feedback, one further standard was removed resulting in a total of 31 Quality Standards being retained.

### Phase 3: Delphi process

To assess acceptability and face validity, a two-stage modified Delphi consultation process was carried out. The Delphi method is a feasible and recognised method of determining the degree of consensus among experts on a given issue [13,14]. This approach has previously been used to identify a generic set of valid quality indicators for primary mental health care for adults [7]. Selection of participants reflected nationally or locally recognised academic, policy, or service development expertise or interest in the field of child and adolescent mental health in primary care. Potential participants were identified by searching for published papers, documents and websites on child and adolescent mental health and by contacting user and professional organisations with an interest in child and adolescent mental health and local Primary Care Trusts. Participants who accepted our invitation (n = 91) were grouped into six panels based on expertise, background, and potential panel size:

1. General practitioners (9 of 13 participants who agreed to participate completed both stages of the Delphi survey).

2. Other community-based professionals (11 of 19 participants completed both stages) - health visitors and practice nurses, practice managers, community paediatricians and members of professional organisations.

3. Child and adolescent psychiatrists (8 of 9 participants completed both stages).

4. Other CAMHS professionals (11 of 24 participants completed both stages) – members of multi-disciplinary teams, psychologists, primary mental health workers, and CAMHS managers and commissioners.

5. Public health and policy specialists (10 of 13 participants completed both stages).

6. Parents and representatives from voluntary organisations (6 of 13 participants completed both stages).

The Delphi consultation process involved a two round electronic/postal questionnaire survey. In the first round, participants were asked to rate: 1) the importance of each of the 31 Quality Standards and 2) how well each Quality Indicator measured its corresponding Quality Standard (on scales of 1–9). Participants were also given the opportunity to provide comments. Non-responders were sent two reminders. Following the first round, the median importance score for each Quality Standard was calculated for each panel. These scores were presented in the second round Delphi questionnaire together with each panellist’s individual score for each Standard. Therefore, each panellist received an individualised second Delphi questionnaire with their panel’s median score and their own previous score for each of the 31 Quality Standards. In the second round, panellists were asked to: 1) re-rate the importance of each Quality Standard and 2) rate how feasible each Quality Indicator was to implement in practice. Hence each Quality Standard was rated on two occasions whereas the corresponding Quality Indicator (which were generated in Phase 2) was assessed for measurement and feasibility on one occasion during the Delphi consultation process.

### Data analysis

As the number of respondents in the Parents and Voluntary Organisations panel was below the requisite number of panellists for a Delphi survey [15], quantitative analyses were only conducted for the remaining five panels.

1) Level of Rating: Panel median importance scores were calculated for Quality Standards and those with a median score of ≥8 were retained. Previous research has found that higher cut-off points (generally, scores that are 8 and above) are associated with a greater level of reproducibility and reliability [16].

2) Consensus: Strict RAND appropriateness method criteria were used to assess agreement, that is: 80% or more of the panellists need to rate the Standard within the 3-point region that contains the median score in order to achieve consensus [15,17]. For example, the three point range for a median importance score of 8 is 7–9. Thus, in a panel of 11 individuals, a minimum of nine panellists have to score the Standard between 7–9 in order to have a consensus median score of 8 (i.e. 80% of the panel score the Standard within the three point range of the median).

For each panel, Quality Standards were identified as important if: (1) they had a median importance score of ≥8 AND (2) there was 80% consensus for the median within the panel. This indicates that, for each panel, the Quality Standard is both rated highly important and that there is consensus on importance within the panel. The final Quality Standards met these criteria across all five panels.

### Phase 4: Piloting the final quality standards

The final set of Quality Standards was piloted with parents at two GP practices to assess their feasibility in practice and to ascertain test-retest reliability. Parents were approached in waiting rooms and asked to rate the importance of the Quality Standards. The scale ranged from: strongly agree, agree, neither agree nor disagree, disagree and strongly disagree. Fifty two parents completed the initial questionnaire and were sent another copy of the questionnaire, either electronically or through the post, after a period of two weeks. Twenty parents completed the second questionnaire. For analyses, the scale was scored from 1–5 (ranging from strongly agree to strongly disagree). Median scores at each time point and intra-class correlations for each Quality Standard were calculated to assess test-retest reliability.

## Results

### Phase 3: Delphi process

#### Quality standards

All five panels agreed that 10 of the 31 Quality Standards were important (see Table 1). The final 10 Quality Standards reflected all four conceptual domains - practice level (one statement reflecting confidentiality), consultation factors (five statements reflecting knowledge, awareness and communication), health visitors (two statements reflecting attitude and continuity of care) and further services (two statements reflecting access and referral).

**Table 1:** **General Practice Standards: Child and Adolescent Mental Health (GPS: CAMH) - Final 10 Quality Standards and corresponding Quality Indicators **

	**Domain**	**Quality Standard**
		**Quality Indicator**
1	Practice (confidentiality)	QS: **GP surgeries should ensure that young people are aware of their policy on confidentiality**
		QI: Does the practice advertise its policy on confidentiality (e.g. posters, leaflets, newsletters, practice website)?
2	Consultation (knowledge)	QS: **GPs’ knowledge about child and adolescent emotional and behavioural health should remain up to date**
		QI: One doctor at the practice should be the lead for work with children and young people
3	Consultation (awareness)	QS: **GPs should be aware that parents may need help and advice in managing and coping with their child’s emotional or behavioural difficulties**
		QI: GPs should direct parents to information for further advice and to support services
4	Consultation (communication)	QS: **GPs should communicate effectively with both parents and children about the child’s emotional and behavioural health**
		QI: Survey of parents and children – do they feel the GP communicates well with them about child and adolescent mental health issues?
5	Consultation (communication)	QS: **GPs should give parents time to talk during the consultation**
		QI: Survey of parents and children – do you feel that the GP gave you time to talk during the consultation?
6	Consultation (communication)	QS: **GPs should be able to communicate with children and young people effectively and build good relationships with them**
		QI: Survey of children and young people – to obtain their opinion about their experience of speaking to the GP and their relationship with the GP
7	Health Visitors (continuity of care)	QS: **Parents should have the choice to see the same Health Visitor in order to establish a relationship where concerns about a child’s emotional or behavioural health can be discussed**
		QI: Survey of parents – have you been able to see the same Health Visitor each time?
8	Health Visitors (attitude)	QS: **Health Visitors should be non-judgemental when listening to parents’ concerns about their child’s emotional or behavioural health**
		QI: Survey of parents – did you feel your Health Visitor was non-judgemental when you spoke about your concerns?
9	Further services (access and referral)	QS: **If appropriate, and if they are unable to provide the help required, GPs should respond to parental requests for help for their child’s emotional or behavioural difficulties by making a prompt referral for further advice**
		QI: Audit the GP practice – Are referrals made to CAMHS and related services? What is the time interval between the appointment and making the referral?
10	Further services (referral)	QS: **GPs should explain the process of a referral and waiting period so that parents feel adequately informed**
		QI: Survey of parents of referred children– did you feel the referral process was adequately explained to you?

© 2012 Sayal et al.

QS: Quality Standard; QI: Quality Indicator.

In total, the GP panel rated 12 statements as being important (i.e. median importance score of ≥8 and 80% consensus for the median within the panel). In contrast, the other four panels rated between 25 and 27 statements as important and agreed with each other that 21 of these statements were important. In relation to these 21 statements, the GP panel median importance score was 9 for one statement (*‘GP surgeries should ensure that young people are aware of their policy on confidentiality’*), 8 for nine statements, 7 for ten statements, and 6 for one statement (‘*GPs should offer parents support and advice on managing difficulties during the waiting period following a referral’*). All these statements achieved consensus within the GP panel.

#### Quality indicators

The corresponding Quality Indicator for each of the final 10 Quality Standards are presented in the Table 1. Quality Indicators were assessed differently in each round (respectively, how well does it measure the Quality Standard and how feasible is it to implement). For both types of assessment (measurement and feasibility), all five panels gave a median score of 7 or above to three of the indicators related to the final ten Quality Standards. These were the indicators reflecting confidentiality, time to talk during the consultation, and should be able to communicate effectively (numbers 1, 5 & 6 in the Table 1). All of the feasibility indicators received a score of 6 or above from each panel. In contrast, three of the indicators received a low median score for measurement from at least one panel. These were the indicators reflecting: GP knowledge (GP panel score 4; Other CAMHS professionals panel score 5), GP awareness (Public Health and Policy panel score 5) and prompt referral (Psychiatrist panel score 5).

### Phase 4: Piloting the quality standards

At both time points, parents in GP practices strongly agreed that nine of the Quality Standards were important (median score of 1). The only exception was for the statement *‘GP surgeries should ensure that young people are aware of their policy on confidentiality’* which received a median score of 2 (agree). Written comments suggested that some parents felt that they should always be involved in the child’s treatment. Intra-class correlations for each Quality Standard were high across the two time points, ranging from 0.66 to 0.95.

## Discussion

Based on parental views about which factors act as barriers and facilitators to seeking help on behalf of their child and their views about appropriate standards of care, we have developed a set of measurable Quality Standards for child and adolescent mental health in primary care. Each Quality Standard comprised a descriptive statement consisting of an element reflecting high-quality care. These Quality Standards were developed collaboratively with parents/caregivers and were further refined following feedback from other parents and a wide range of professionals who work with children, adolescents, and families. Following the Delphi consultation process, there was consensus on the importance of 10 of 31 parent-derived Quality Standards. Overall, the GP panel were the most conservative, rating the fewest number of Quality Standards as important. This discrepancy between professional and user/carer views has also been found in relation to quality priorities for primary mental healthcare for adults [18,19]. In the present study, the discrepancy mainly reflected the receipt of slightly lower importance scores from the GP panel – ten statements received median importance scores of 7 from the GP panel and ≥8 from the other panels.

The final set of Quality Standards comprised a variety of factors, particularly process measures which are increasingly regarded as an important facet of assessing quality of care [20]. Some of the standards reflect important generic issues within primary care such as communication, professionals’ attitudes, continuity of care, concerns being taken seriously and the likelihood of receiving appropriate and timely help [21]. Relationship continuity remains a high priority for patients and caregivers across all aspects of primary care [22]. The need for prompt referral for more specialist input if the GP was unable to help and for support during the interim period was also highlighted. Although elicited from parents, two Quality Standards were directly related to the child's subjective experience of communication and confidentiality within primary care. Interestingly, the statement on awareness of confidentiality for young people received the highest importance score from the GP panel but weaker endorsement from the parents who participated in the piloting phase at the GP practices. This might reflect parental concerns about the shift in the initiation of help-seeking from the parent to the child with increasing age. Other Quality Standards reflected GP knowledge and awareness of child emotional and behavioural difficulties. In order to be more responsive to families’ concerns, primary healthcare professionals and services should be aware of potential barriers to seeking help and ways of breaking down these barriers [9]. The use of these standards provides a possible means of increasing professionals' awareness of parental views and enabling them to recognise and appropriately respond to these difficulties. The standards are also potentially useful in providing information to service commissioners about the quality of child and adolescent mental healthcare in primary care.

### Strengths and limitations

The study used an iterative approach involving several phases to obtain a wide and comprehensive range of opinions in order to ensure that the final Quality Standards were a valid reflection of parental and professional views. Overall, there was a good response to the Delphi survey, suggesting that the final Quality Standards are a reliable reflection of views. However, dropout rates over the two rounds were relatively high for two of the panels: the other CAMHS professionals and Parents and Voluntary organisations panels. Participants for the latter panel were the hardest to recruit despite initial invitations to a large number of organisations. For future research, conducting interviews or focus groups might be better for recruiting a higher number of respondents and minimising attrition rates. In terms of methodology, the feasibility and the validity of the proposed Quality Indicators were only assessed once during the Delphi survey and should be considered less robust than the corresponding Quality Standard. The study was conducted in one geographical area and the generalisability of the findings to other countries and healthcare settings is uncertain. Finally, as the study focussed on the perspective of the parent/caregiver, it is not known how these findings might concord with older children's or professionals' views about markers of quality of primary mental healthcare. For example, the final standards did not include items on the detection of mental health problems [3] or the provision of evidence-based interventions.

### Clinical and research implications

This study reiterates the importance of user involvement, in this case parents, in research to assist initiatives to improve health service delivery. As well as identifying barriers to accessing care, this study has developed ten Quality Standards that could be utilised to improve the quality of care for children and young people. For the final set of standards, there was consensus across a wide range of stakeholders that these reflected quality. Therefore, these statements are useful markers to assess quality of care. The importance of developing these quality standards also reflects their potential to improve the quality of patient experience, increase the likelihood of parental presentation of emotional and behavioural concerns and receipt of appropriate interventions, and improve outcomes. The Quality Standards highlight areas that should be prioritised for quality improvement programmes, service delivery and organisation, and future intervention research that aim to improve access to services and outcomes for children. The outputs could be useful for commissioners and managers of primary and secondary care child health services in carrying out local needs assessments. The use of routine measures in primary care could play a role in raising awareness of child mental health issues in an everyday setting. Additionally, they may also improve the detection of child mental health problems which, in turn, could improve outcomes for affected children and for their families.

Our findings highlight the importance of GPs being sufficiently knowledgeable about emotional and behavioural problems in children and young people and being able to communicate effectively with children, young people and parents about these problems. GPs should be aware that some parents may need support and advice on how to deal with their child’s behaviour and, where appropriate, they should inform parents about treatment options and their availability. The findings raise the question of whether current GP and health visitor training on child and adolescent mental health and effective ways of communicating with parents, young people and children about sensitive issues should be enhanced. The findings also highlight the need for a good system of communication between primary and secondary health care services. Such improvements in communication would ensure that GPs have accurate information to share with parents about both the procedures involved with a referral and the length of time it might take before interventions can commence.

In terms of future research, as the utility of the instrument still needs to be demonstrated in practice, wider piloting and benchmarking of these standards across a range of GP practices is required. This should be accompanied by further refinement of the most appropriate indicator to best measure each standard. A better understanding of reasons for the discrepancies between GP and other stakeholders' views about the initial set of Quality Standards and the weaker parental support for the importance of children's awareness about policies relating to confidentiality also merits further research.

## Conclusions

Based on parents’ experiences, the generated Quality Standards to assess mental healthcare for children and adolescents seen within primary care services provide a measure that can be used at the individual clinician or practice level. These Quality Standards could be used to improve the quality of care for child and adolescent mental health problems within primary care.

## Competing interests

The authors declare that they have no competing interests.

## Authors’ contributions

We confirm that all authors fulfil the criteria for authorship. All authors contributed core ideas and to writing the paper. KS had the original concept and designed the study, supervised data collection and managed the study, participated in data acquisition, analysis and interpretation, and drafted and finalised the paper. MAm, SR & CC recruited the sample, participated in data acquisition, analysis and interpretation, and helped to draft the manuscript. MAs, CD, AT, ES participated in the conception and design of the study, interpretation of the findings, and revising the paper critically for important intellectual content. All authors read and approved the final manuscript.

## Pre-publication history

The pre-publication history for this paper can be accessed here:

http://www.biomedcentral.com/1471-2296/13/51/prepub
